# Anesthetic management of giant thyroid tumor with cardiac comorbidity causing tracheal compression: a case report

**DOI:** 10.1186/s13256-025-05707-z

**Published:** 2025-12-29

**Authors:** Haikun Zhang, Jinxiang Yu, Chengcheng Chen, Le Cao, Pengcheng Ma, Lifeng Jia, Nianliang Zhang, Tao Zhao

**Affiliations:** 1 School of Anesthesiology, Shandong Second Medical University, Weifang, 261053 Shandong China; 2 Department of Radiology, People’s Hospital of Rizhao, Rizhao, 276826 Shandong China; 3 Shandong Provincial Key Medical and Health Laboratory of Perioperative Precise Anesthesia and Organ Protection Mechanism Research, Rizhao Key Laboratory of Basic Research On Anesthesia and Respiratory Intensive Care, Department of Anesthesiology, People’s Hospital of Rizhao, Rizhao, 276826 Shandong China

**Keywords:** Thyroid tumor, Tracheal compression, Cardiac disease, Awake intubation, Multidisciplinary collaboration

## Abstract

**Background:**

This case report described the entire process of a 72-year-old Chinese woman undergoing resection of a giant thyroid tumor. The novelty of this case report lies in its emphasis on the crucial role of anesthetic management for giant thyroid tumors, particularly in patients with concurrent cardiac comorbidities.

**Case presentation:**

We present a 72-year-old Chinese female with a giant thyroid tumor caused respiratory compromise due to tracheal compression, complicated by atrial septal defect. She required general anesthesia for tumor resection. A multidisciplinary team developed critical contingency strategies: (1) awake endotracheal intubation under direct laryngoscopy, (2) a remimazolam/sufentanil combination for procedural tolerance, (3) improved tracheal catheter preparation, and (4) surgical tracheostomy readiness. Anesthesia maintenance was achieved with sevoflurane, supplemented by sufentanil and remifentanil for multimodal analgesia and vecuronium/mivacurium neuromuscular blockade. Successful tumor resection achieved complete decompression, with no postoperative complications documented during 30-day follow-up.

**Conclusions:**

This case demonstrates that meticulous interdisciplinary communication and structured perioperative protocols form the cornerstone of safe anesthesia practice for patients with dual pathology of airway compromise and cardiac comorbidities, providing crucial insights for managing such complex clinical scenarios.

## Background

Anesthetic management of giant thyroid neoplasms with concurrent cardiac comorbidities and tracheal compression poses significant clinical challenges, as patients often present with tracheal stenosis secondary to mechanical compression by the tumor mass [1]. The coexisting cardiac pathology significantly increases risks of compromised circulatory compensatory mechanisms. The induction phase carries dual risks of complete airway obstruction from tracheal collapse and hemodynamic instability due to acute cardiac load fluctuations [2]. While awake fiberoptic bronchoscopy-guided intubation remains the international standard, the diminished oxygen reserve in cardiac patients necessitates precision-targeted oxygenation strategies. This case report provides practical insights into managing complex anesthesia scenarios through the paradigm of a congenital heart disease patient with giant thyroid neoplasm-induced tracheal compression.

## Case presentation

A 72-year-old Chinese female (52 kg) incidentally discovered a left thyroid mass 30 years ago. At that time, she experienced no discomfort, and the mass did not impact her daily life. The patient’s asymptomatic presentation fostered a false sense of security, compounded by procedure-related apprehension characteristic of the older population. She did not seek any specific treatment for it. However, over the past 2 years, she developed progressive dyspnea and dysphagia, which worsened in the last month, prompting her decision to undergo surgical treatment. No contributory family history was identified. Her medical history included congenital heart disease, hypertension, and coronary artery disease, with prior cholecystectomy and splenectomy in 2016 for hereditary spherocytosis. Physical examination revealed normal vital signs with grade III left thyroid enlargement accompanied by stridor and odynophagia, without audible cardiovascular bruits. The massive tumor caused restricted neck mobility with rightward tracheal deviation, Mallampati class III airway, and three-finger interincisor distance. Visual laryngoscopy demonstrated normal vocal cord mobility and configuration. Electrocardiography demonstrated atrial fibrillation with rapid ventricular response (113 bpm) and nonspecific ST-T wave abnormalities. Echocardiography revealed biatrial enlargement, moderate mitral and tricuspid regurgitation with mild aortic regurgitation, atrial septal defect, atrial-level left-to-right shunt flow, pulmonary hypertension (estimated pulmonary artery systolic pressure [PASP] 42 mmHg), and inferior vena cava collapsibility index > 50%. During the examination, the patient presented with an irregular heart rhythm and was classified as New York Heart Association (NYHA) class II for heart function.

**Fig. 1:**
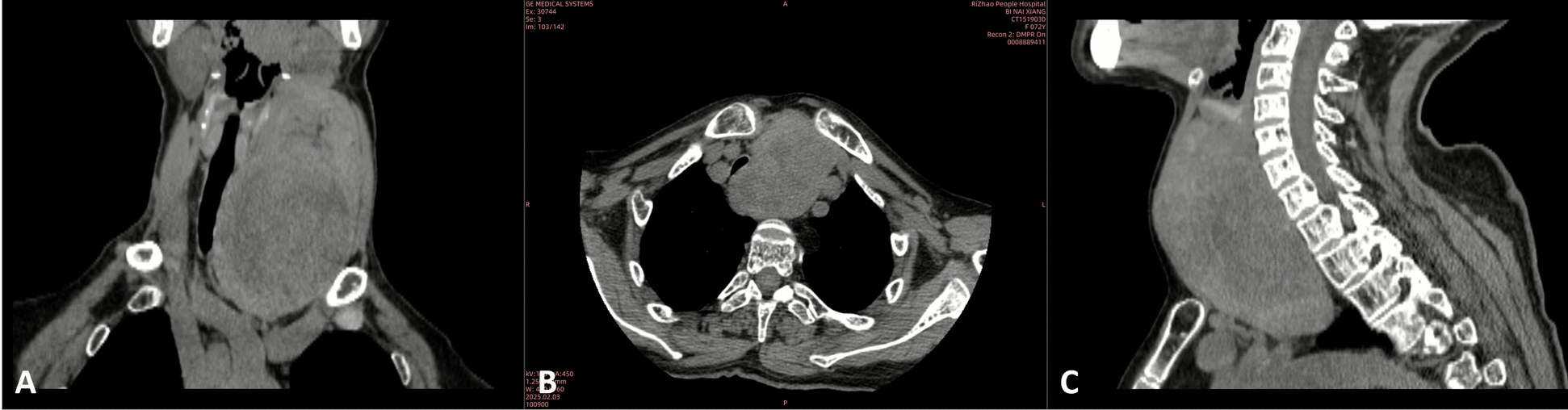
Contrast-enhanced CT images of the neck demonstrating a large mass causing compression of the surrounding structures in different planes, including coronal (**A**), axial (**B**), and sagittal (**C**) views

**Fig. 2:**
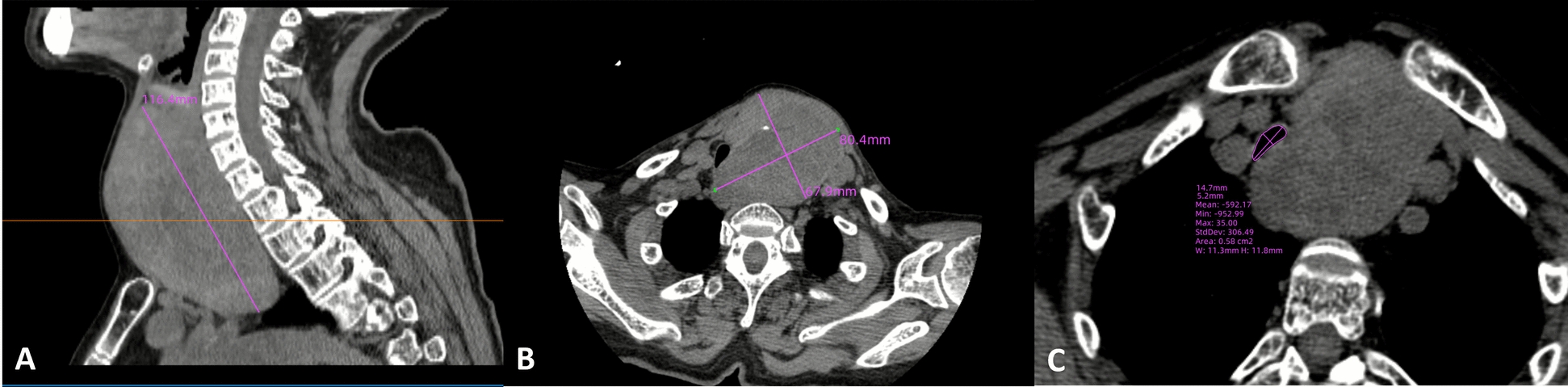
Thyroid mass measurements and airway compression. Quantitative assessment of thyroid mass and airway compression. Sagittal view showing the tumor long-axis length of 116.4 mm (**A**); axial view demonstrating tumor dimensions of 67.9 × 80.4 mm (**B**); and axial view at the narrowest level showing a tracheal cross-sectional area of 0.58 cm^2^ with a minimal lumen diameter of 5.2 mm (**C**)

**Fig. 3:**
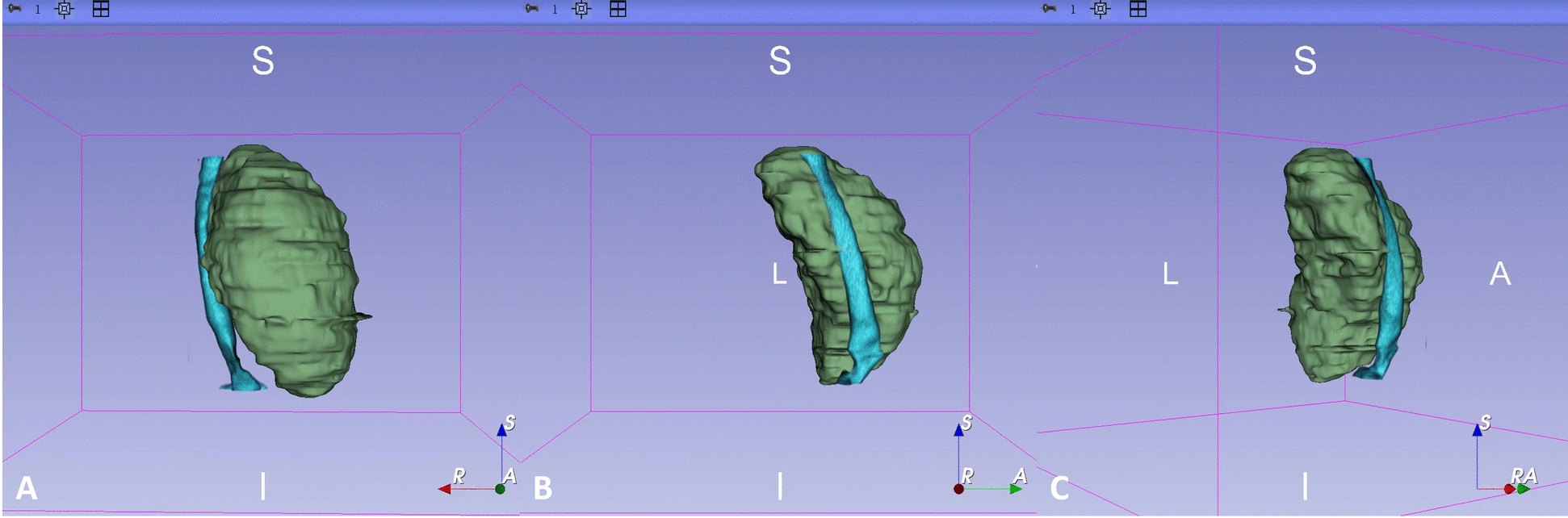
Three-dimensional computed tomography reconstruction illustrating the anatomical spatial relationship between the thyroid neoplasm and upper airway. The tumor mass effect (green overlay) causes anterior compression of the tracheal wall (airway lumen highlighted in blue), observed from three different perspectives: anteroposterior projection (**A**), right lateral projection (**B**), and right posterolateral oblique projection (**C**)

The patient maintained euthyroid status with serum free thyroxine, free triiodothyronine, and thyroid-stimulating hormone levels within normal reference ranges. Color Doppler ultrasonography demonstrated a left thyroid nodule with benign characteristics. Contrast-enhanced computed tomography (CT) images were provided in the coronal, axial, and sagittal planes (Fig. 1). It confirmed thyroid enlargement, with sagittal imaging demonstrating a mass with a long axis of 116.4 mm and axial imaging revealing a lesion measuring 67.9 × 80.4 mm, causing severe extrinsic compression of the trachea (Fig. 2). At the level of maximal compression, the tracheal lumen demonstrated a minimal cross-sectional area of 0.58 cm^2^; the narrowest tracheal lumen diameter at the compressed site was 5.2 mm (Fig. 2), with gradual restoration to normal caliber distally; and no evidence of tracheal wall infiltration was observed. Three-dimensional CT reconstruction was performed to better delineate the spatial relationship between the mass and trachea (Fig. 3).

Preoperative workup was fully completed 24 hours prior to the procedure. During the preoperative day afternoon session, a multidisciplinary team (MDT) consultation involving general surgery, anesthesiology, otorhinolaryngology, cardiology, and thoracic surgery formulated comprehensive surgical and contingency protocols, with a special focus on peri-induction critical events, including difficult airway scenarios and cardiovascular crises. Emergency strategy includes the establishment of a surgical airway. The patient declined prophylactic tracheostomy. Therefore, the procedure was primarily considered under the following circumstances: preoperative acute respiratory distress; preoperative acute upper airway obstruction due to bilateral recurrent laryngeal nerve involvement; failed reintubation; intraoperative identification of a very high risk of bilateral recurrent laryngeal nerve injury; or evident postoperative tracheal collapse, malacia, or respiratory failure following extubation. The tumor was situated posterolaterally on the left aspect of the trachea, causing significant compression. Consequently, we used ultrasound to precisely delineate the tracheal course in preparation for potential emergency tracheostomy. The measures under consideration include high-frequency jet ventilation and extracorporeal membrane oxygenation (ECMO). Preoperative pharmacological optimization included oral esmolol administration for heart rate control.

**Fig. 4:**
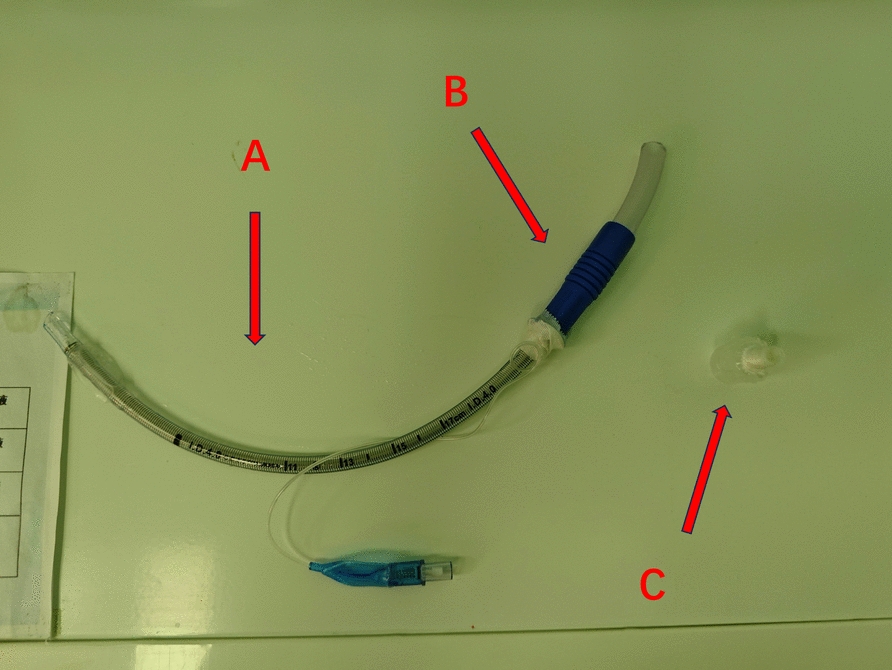
The customized extended-length airway. The customized extended-length airway device comprised three key components: **A** a reinforced endotracheal tube with a 4.0 mm internal diameter (arrow), **B** a 5 cm sterile suction catheter tip adapter secured with medical-grade adhesive tape (arrow), and **C** a removed proximal connector (arrow)

**Fig. 5:**
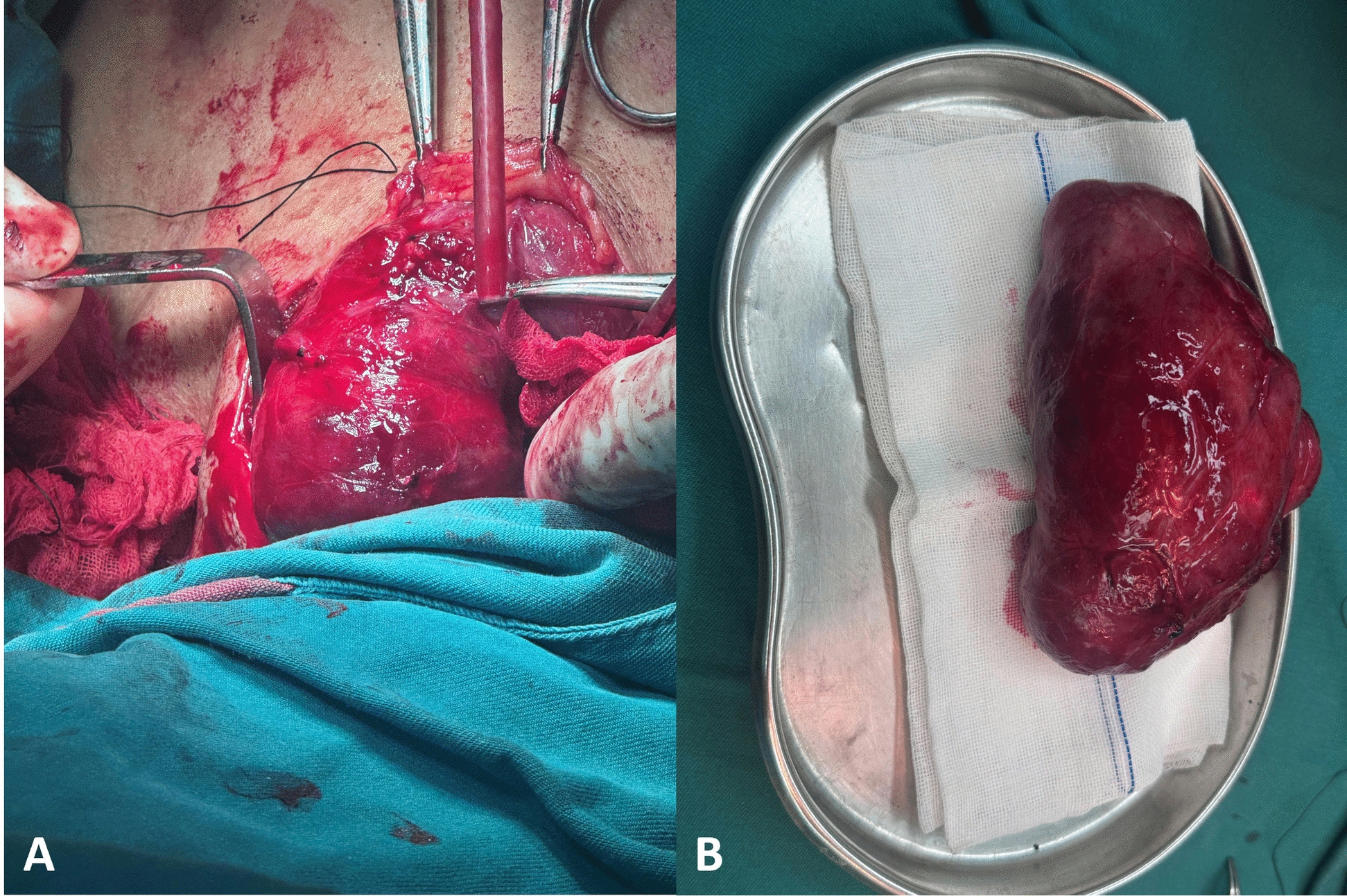
Thyroid mass: Representative intraoperative image (**A**) showing the exposed thyroid mass during surgical resection, and postoperative image (**B**) demonstrating complete removal of the mass after dissection

Upon arrival in the operating theater at 8:10 a.m., standard monitoring revealed the following baseline parameters: blood pressure 116/72 mmHg, heart rate 70 bpm, and oxygen saturation (SpO_2_) 99%. We initially selected a 6.0 mm endotracheal tube (ETT) as the primary airway device. Considering the severe airway narrowing, we were concerned that the external diameter of the 6.0 mm ETT, along with its cuff, would not pass smoothly through the narrowest part of the airway. Forcing it through under resistance could have potentially caused friction against the tracheal mucosa, leading to edema, bleeding, or even tearing. A 4.0 mm ETT with a customized length extension was prepared as a plan B because its external diameter (with cuff deflated) does not exceed 5.0 mm: We used medical tape to connect the 4.0 mm ETT (removing the connector) to the suction joint and glued it together to form an extended tracheal tube (Fig. 4). Awake intubation was performed under monitored anesthesia care with 10 μg sufentanil and 10 mg remimazolam at 8:30 a.m. Following glottic visualization using the visual laryngoscope, 3 mL of 2% lidocaine was sprayed to the supraglottic area, supplemented by 2 mL of transtracheal instillation for complete airway anesthesia. Successful intubation was achieved with the 6.0 mm tube positioned at 23 cm at the incisors, confirmed by end-respiratory carbon dioxide waveform and absence of an audible air leak. Post-intubation management included 8 mg vecuronium for neuromuscular blockade, 150 mg hydrocortisone sodium succinate for airway edema prophylaxis, and sevoflurane (minimum alveolar concentration 1.0) for anesthesia maintenance. Hemodynamic stability was maintained with a 10 mg ephedrine bolus and esmolol 200 mg on standby. The surgical incision was performed at precisely 8:50 a.m. Pre-incision analgesia was ensured with 30 μg sufentanil, followed by remifentanil target-controlled infusion at 0.1–0.3 μg/kg/min. Metaraminol infusion stabilized hemodynamics within 15% of baseline values. Neuromuscular blockade was maintained with mivacurium following tumor dissection. The 2.5-hour procedure concluded with complete tumor resection (Fig. 5) without airway compromise. Fluid management included a 1500 mL crystalloid infusion, 200 mL of estimated blood loss, and 650 mL of urine output. Postoperative extubation following standardized emergence protocol revealed intact respiratory function, with uneventful post-anesthesia care unit (PACU) transition.

On extubation day, the patient maintained stable spontaneous respiration without stridor, tracheal collapse, or tracheomalacia. By postoperative day 7, bilateral vocal cord mobility was normal with no hoarseness or aspiration. At 30-day follow-up, the patient showed significant quality-of-life improvement, with complete resolution of preoperative dysphagia and neck compression symptoms. She expressed particular satisfaction with the airway management approach, as it avoided tracheostomy.

## Discussion and conclusions

While giant thyroid neoplasms are clinically prevalent, their anesthetic management requires meticulous attention, particularly when complicated by tracheal compression and concurrent cardiopulmonary comorbidities. The primary challenge fundamentally stems from tumor-induced anatomical distortion of the airway architecture, significantly compromising airway management during induction and intubation phases [3]. In patients with thyroid neoplasms, preoperative respiratory compromise secondary to tracheal compression may necessitate emergent surgical airway establishment to secure ventilation [4]. Chronic tumor compression may exacerbate preexisting cardiac comorbidities, potentiating hemodynamic instability through mechanical interference with mediastinal structures. While advanced imaging (CT/magnetic resonance imaging [MRI]) delineates tracheal stenosis parameters, anatomical distortion in thyroid pathology often renders conventional laryngoscopy ineffective. This necessitates integrated airway assessment with contingency planning for fiberoptic bronchoscopy (FOB)-guided intubation [5].

This case involved a giant thyroid neoplasm causing tracheal compression and respiratory compromise, with resultant airway anatomical distortion that significantly increased intubation complexity. Preoperative evaluation required precise characterization of the tumor’s pathological features, quantitative assessment of tracheal compression, and comprehensive cardiopulmonary functional analysis. Neck CT was used to demonstrate tracheal stenosis, the direction of deviation, and the inner diameter at the narrowest point. When conditions permit, CT three-dimensional reconstruction can accurately quantify the degree, location, and anatomical relationship between tracheal stenosis and peripheral blood vessels, providing key preoperative mapping for the spatial relationship between tumors and adjacent vascular structures. The coexistence of congenital heart disease, pulmonary hypertension, and atrial fibrillation necessitated differential diagnosis of dyspnea etiology using echocardiographic strain analysis. A tiered response team comprising three senior anesthesiologists and two surgeons maintained direct visual contact; they were equipped with immediate access to advanced airway rescue devices (including emergency cricothyrotomy kits and jet ventilation systems). ECMO was adopted when necessary.

In patients with tracheal stenosis secondary to extrinsic compression, rapid sequence induction carries substantial risk of complete airway obstruction due to loss of structural support following neuromuscular blockade [6]. Given executive capacity limitations in ECMO, awake tracheal intubation (ATI) with preserved spontaneous ventilation was prioritized to mitigate hemodynamic fluctuations, which is particularly critical in patients with preexisting cardiopulmonary compromise [7]. Fiberoptic bronchoscopy (FOB)-guided intubation under conscious sedation remains the gold standard for critical tracheal stenosis, enabling direct visualization to navigate beyond compressive lesions while minimizing hemodynamic stress through precise glottic localization [8]. Although video laryngoscopy provided adequate glottic visualization in this case, the hybrid approach combining FOB with optical devices may optimize difficult airway management in similar scenarios by leveraging multimodal visualization advantages. Reasonable modifications to the endotracheal tube may overcome anatomical constraints while preventing gas leakage complications associated with conventional tubes requiring pharyngeal packing [9]. The improved tracheal catheter may circumvent this risk [10, 11]. While not implemented in this case, the preprepared modified airway device represented an innovative contingency strategy, highlighting the importance of adaptive equipment preparation in complex airway scenarios. However, the 4.0 mm ETT was not the optimal choice for ventilation in this case. While it passed through the narrowed airway without difficulty, we were concerned that it would not reach the safe position above the tracheal ridge. Therefore, we made an additional extension to the ETT. We do not advocate the use of improved tracheal catheter for mechanical ventilation in normal circumstances, as the risk factors sometimes outweigh the potential benefits. The loss and displacement of the catheter, along with barotrauma caused by passive breathing, are inevitable risks associated with improved tracheal catheter. Currently, devices such as Ventrain or Tritube may offer greater convenience for patients with airway narrowing.

Successful ATI requires a multimodal approach encompassing sedation, topical anesthesia, oxygen supplement, and ergonomic positioning and complemented by effective therapeutic communication [12]. The protocol employed remimazolam with sufentanil and lidocaine to achieve the optimal balance between procedural comfort and hemodynamic stability during ATI. Remimazolam, an ultra-short-acting benzodiazepine with organ-independent metabolism, demonstrates a superior hemodynamic profile compared with midazolam while avoiding prolonged sedation [13]. A randomized trial demonstrated remimazolam/remifentanil combination achieved tighter hemodynamic control (mean arterial pressure fluctuation < 10% baseline) with significantly higher intraoperative neuromonitoring success rates versus propofol-based regimens [14]. Dexmedetomidine, a selective α2 agonist, shares sedative and analgesic properties with remimazolam while preserving respiratory drive. Despite its widespread clinical adoption, a multicenter randomized controlled trial (RCT) revealed superior amnestic effects with remimazolam versus dexmedetomidine and shorter intubation durations [15]. However, adjusting the position of the operating table can help relieve tumor compression and ensure the optimal “comfort” of the patient’s breathing and hemodynamics.

Intraoperative monitoring is equally critical. In addition to the invasive arterial pressure monitoring that we performed, transesophageal echocardiography (TEE) is a crucial method for assessing cardiopulmonary reserve. It allows for monitoring of cardiac output and volume status through different imaging planes. If acute cardiopulmonary compensatory dysfunction occurs during the procedure, ECMO must be initiated. This requires preoperative confirmation of access routes and preparation to ensure free access to the inguinal region during the procedure, facilitating preparation for extracorporeal circulation.

Additionally, a precise assessment of the timing for extubation is crucial. Patients with heart disease often have reduced tolerance to extubation stress [16]. Furthermore, after the relief of tumor compression, there is a risk of reintubation due to insufficient elastic recoil or postoperative edema in the trachea. In our case, the patient did not show a significant tendency toward tracheal collapse and, after regaining spontaneous breathing, followed commands, demonstrating adequate tidal volume. Following a safe extubation, the airway remained patent, and there were no particular hemodynamic fluctuations.

The above discussion is based on the majority of patient cases, and it is always necessary to tailor management to individual circumstances. We maintain agreement with what has already been achieved and adopt a proactive attitude toward continuous improvement in areas yet to be addressed.

Anesthetic management of giant thyroid neoplasms with cardiopulmonary comorbidities requires MDT collaboration. Awake fiberoptic intubation provides an enhanced safety profile through preserved upper airway tone and continuous respiratory drive maintenance. Real-time multimodal monitoring enables proactive intervention compared with conventional monitoring. In summary, a comprehensive preoperative plan and close collaboration among multidisciplinary teams are crucial for ensuring the safety of such patients.

## Data Availability

Not applicable, but appropriate materials are available upon request.

## Abbreviations

NYHA: New York Heart Association
MDT: Multidisciplinary team
ECMO: Extracorporeal membrane oxygenation
ETT: Endotracheal tube
ATI: Awake tracheal intubation
FOB: Fiberoptic bronchoscopy
TEE: Transesophageal echocardiography
